# Low-Dose Radiotherapy Leads to a Systemic Anti-Inflammatory Shift in the Pre-Clinical K/BxN Serum Transfer Model and Reduces Osteoarthritic Pain in Patients

**DOI:** 10.3389/fimmu.2021.777792

**Published:** 2022-01-03

**Authors:** Thomas Weissmann, Michael Rückert, Jian-Guo Zhou, Michaela Seeling, Sebastian Lettmaier, Anna-Jasmina Donaubauer, Falk Nimmerjahn, Oliver J. Ott, Markus Hecht, Florian Putz, Rainer Fietkau, Benjamin Frey, Udo S. Gaipl, Lisa Deloch

**Affiliations:** ^1^ Department of Radiation Oncology, Universitätsklinikum Erlangen, Friedrich-Alexander-Universität Erlangen-Nürnberg, Erlangen, Germany; ^2^ Translational Radiobiology, Department of Radiation Oncology, Universitätsklinikum Erlangen, Friedrich-Alexander-Universität Erlangen-Nürnberg, Erlangen, Germany; ^3^ Department of Oncology, The second affiliated Hospital of Zunyi Medical University, Zunyi, China; ^4^ Department of Biology, Institute of Genetics, Friedrich-Alexander-Universitätsklinikum Erlangen, Friedrich-Alexander-Universität Erlangen-Nürnberg, Erlangen, Germany

**Keywords:** low-dose radiotherapy, osteoarthritis, rheumatoid arthritis, mouse model, foot, pain, X-rays, K/BxN serum transfer

## Abstract

Osteoarthritis (OA) is the leading degenerative joint disease in the western world and leads, if left untreated, to a progressive deterioration of joint functionality, ultimately reducing quality of life. Recent data has shown, that especially OA of the ankle and foot are among the most frequently affected regions. Current research in OA points towards a complex involvement of various cell and tissue types, often accompanied by inflammation. Low-dose radiotherapy (LDRT) is widely used for the treatment of degenerative and inflammatory diseases. While the reported analgesic effects are well known, the underlying molecular mechanisms are only poorly understood. We therefore correlated a clinical approach, looking at pain reduction in 196 patients treated with LDRT with a pre-clinical approach, utilizing the K/BxN serum transfer mouse model using flow cytometry and multiplex ELISA for analysis. While an improvement of symptoms in the majority of patients was found, patients suffering from symptoms within the tarsi transversa show a significantly lower level of improvement. Further, a significant impact of therapy success was detected depending on whether only one or both feet were affected. Further, patients of younger age showed a significantly better outcome than older ones while needing fewer treatment series. When looking on a cellular level within the mouse model, a systemic alteration of immune cells namely a shift from CD8+ to CD4+ T cells and reduced numbers of DCs was observed. A general reduction of inflammatory cytokines was detected, with significant alterations in IL-4 and IL-17 levels, all of which could potentially be responsible for the highly effective clinical improvement in patients. Taken together our data indicate that LDRT can be regarded as a highly effective treatment option for patients suffering from OA of the foot and ankle, in terms of analgesic effects, especially in younger patients. Furthermore, the observed effects are mediated by an interplay of cellular and soluble immune factors, as observed in the K/BxN serum transfer model. With this interdisciplinary approach we aim to encourage the usage of LDRT as an additive treatment strategy not only as a last resort, but also earlier in the course of disease.

## Introduction

Osteoarthritis (OA) as the leading degenerative joint disease leads, during the course of the disease, to a reduction in functionality, consequently resulting in reduced quality of life. OA in general accounts for approximately 15% off all musculoskeletal consultations in primary care (1). The high prevalence of OA affects 10% of men and 18% of women over 60 years of age (2, 3) thus posing a true socioeconomic burden consuming up to 1.0 – 2.5% of the gross domestic product in the western world due to treatment costs and loss of productivity (4). While the disease seems to undergo a slow but steady progression, its initial occurrence seems to be accompanied by multiple genetic, biological and biomechanical risk factors such as female gender, higher age, obesity, mechanical strain, idiopathic reasons and suffered injuries. While former pathogenesis was understood as a simple mechanical cartilage degradation, the evolved scientific insights describe OA as a complex interaction of different types of tissue and cells. Here, a diverse spectrum of matrix proteases underlying a vast number of pathways is involved, causing changes in cartilage volume, proteoglycan content, cartilage vascularization and cartilage perfusion leading to an involvement and inflammation of the whole joint resulting in a steady deterioration (5, 6).

While the occurrence of OA is, in theory, possible for every joint, recent data substantiate OA of the ankle and foot to be among the most frequent in the lower limbs (7). OA of the foot is most often reported as being mostly found in the metatarsophalangeal joint and the mid foot and less often in the hind foot or ankle. Different studies indicate a higher incidence of OA in abnormal structural status of the foot like toe length abnormalities, wider bone structure, long sesamoids, flat feet or higher arches of the foot (8). Patients with OA of the feet suffer from a variety of symptoms including pain, stiffness, swelling, as well as limited range of motion resulting in a decreased physical function. End-stage OA of the foot can lead to severe deformities that ultimately substantially reduce the mobility of patients.

Treatment strategies for OA of the foot and ankle pose a true challenge due to the complex structure of the entire organ. Therefore, treatment should always be adapted to individual characteristics recommending conservative treatment approaches in early stages and more invasive procedures in advanced stages. Conservative approaches include basic strategies like weight-loss approaches as well as physical therapy and the utilization of ortheses. Drug medication includes a broad variety of substance groups like non-steroidal anti-inflammatory drugs (NSAIDs) and biologics, applied solely or in combinations. Further approaches include the intraarticular injection of glucocorticoids, hyaluronic acid or radiosynoviorthesis (9). Surgery should be regarded as an ultima ratio approach for advanced or end-stage disease. On the other hand, radiotherapy (RT) for benign diseases holds the potential of filling the gap between conservative and more invasive treatment approaches.

Low-dose radiotherapy (LDRT) with X-rays for different degenerative and inflammatory diseases such as enthesiopathies and OA has successfully been applied for decades and is further gaining acceptance by orthopedic specialists and general physicians due to the satisfying results achieved (10–15). Especially patients that are refractory to classical treatment options may benefit from LDRT, as the therapy induces long-lasting pain-relieving effects. Several pre-clinical and clinical studies provide evidence for the effect of LDRT on the immune system and modulation of inflammation (16, 17).

Further effects include osteoimmunological mechanisms affecting bone formation and resorption activity of osteoclasts (18–20), the inflammatory phenotype of macrophages (21–23) or the adhesion of infiltrating immune cells to endothelial cells (24–26) that are most likely responsible for the pain relieving and anti-inflammatory effects (14, 27, 28). Although these progressive scientific insights have significantly diluted criticisms like the major effect being caused by placebo-effects, the underlying molecular mechanisms still need to undergo extensive fundamental research to make the transition from preclinical data to clinical outcome more comprehensible. Placebo-controlled studies are still needed and patient samples cannot always be taken easily from the affected regions. We therefore examined the corresponding regions in a K/BxN serum transfer mouse model in order to investigate LDRT-induced effects at a molecular level. While this mouse model is generally looked at as a model for inflammatory arthritides (29), it also shares similarities with OA, such as a fibrotic synovium releasing inflammatory cytokines (e.g. Interleukin (IL)-1 and tumor necrosis factor-α (TNF-α) (30), activated macrophages and inflammation (5, 31, 32). Additionally, this mouse model also shows signs of cartilage and bone remodeling as well as destruction, signs, that can also be found in OA joints (33) and is a useful tool in order to examine arthritic pain (29, 34). Further, as summarized in (33, 35, 36) there is no ideal murine model that can be regarded as the “gold standard” in OA, as all of the model systems lack some of the properties observed in patients. Additionally, we were able to show in previous studies, that an underlying inflammatory condition is needed for LDRT to be able to modulate inflammation that results in the observed beneficial effects (18, 19). In the present patient study we mainly focused on the analgesic effects of LDRT that, according to our hypothesis, are mediated *via* a modulation of inflammation. We thus opted for the approach to use the K/BxN serum transfer mouse model as an inflammatory model with pathological changes in the joints for pain related processes.

Despite the rising prevalence of OA due to the higher age of the general population, OA of the foot and ankle is rather seldom subject to intensified clinical research. RT of the ankle and foot has been reported only very sporadically presenting only a very limited number of patients (37).

In addition, further pre-clinical research that helps to discover the underlying mechanisms involved in anti-inflammatory and analgesic effects of LDRT is needed. In the present study, we therefore combined clinical and pre-clinical data in order to better understand the underlying mechanisms of LDRT.

## Material and Methods

### Patient Cohort

Between November 2004 and February 2019, 196 (47 men, 149 woman) patients with OA of the foot and ankle, received LDRT at the Department of Radiation Oncology of the Universitätsklinikum Erlangen. The patients were referred by their treating orthopedic specialist. All patients had received several therapies before undergoing LDRT, as an alternative treatment modality. The majority of the patients did not undergo full clinical, rheumatologic diagnostics directly before therapy. In general, patients have been referred to our clinic with a history of chronic degenerative joint disease of the foot and ankle. The categorization of different localizations was carried out by marking the origin of pain before undergoing LDRT. At time of LDRT, the median age of the patients was 65.9 years with a median age of 66.4 for women and 63.7 for men. As pre-clinical studies indicate stronger anti-inflammatory effects with a single dose of 0.5 Gy, most patients underwent LDRT with 0.5 Gy. 177 patients underwent LDRT with a single dose of 0.5 (total dose 3 Gy) and 19 patients underwent LDRT with a single dose of 1.0 Gy (total dose 6 Gy). Furthermore, 24 patients received one series of LDRT, 165 patients received two series and 6 patients underwent three series of LDRT. Only one patient underwent four consecutive series of LDRT. **Table 1** provides a summary of these patient characteristics.

**Table 1:** Overview of patient characteristics.

Patient characteristics	Cohort (N = 196)
**Gender (Number of patients/[%] total cohort)**	
Male	47/24%
Female	149/76%
**Age (Mean ± SD)**	
All patients	65.9 ± 14.5
Male	63.7 ± 12.6
Female	66.4 ± 15.1
**Number of series (Number of patients/[%] total cohort)**	
1	24/12%
2	165/84%
3	6/3%
4	1/0.5%
**Applied single dose (Number of patients/[%] total cohort)**	
0.5 Gy	177/90%
1.0 Gy	19/10%
**Localization (Number of patients/[%] total cohort)**	
Metatarsophalangeal	49/25%
Tarsophalangeal	51/26%
Tarsi transversa	40/20%
Lower ankle	21/11%
Upper ankle	35/18.5%
**Affected side (Number of patients/[%] total cohort)**	
Right	83/42%
Left	73/37%
Both	40/20%

Written informed consent was obtained from all patients before LDRT. In addition, patients provided informed consent for the retrospective analysis of their data, as well as the publication of the data in a pseudonymized manner (ethical approval Ref.169_21 Bc, FootRetroRad trial).

### Treatment

#### Patients

All patients underwent LDRT applied with an orthovoltage technique. LDRT was applied using a Stabilipan machine (180kev, 20 mA, 0,2mm Cu filter, focus skin distance 40 cm) until September 2016. Afterwards a X-Strahl machine was used (180 keV, 10mA, 0,2mm Cu filter, focus skin distance 50 cm). No qualitative differences can be expected concerning radiation quality. The single fields applied for both machines ranged from 6 x 8cm to 10 x 8cm and 10 x 15cm, according to the field needed for the joint. The fields were positioned directly on the affected joint with a determination of the dosage at the middle of the joint. One radiotherapy series consisted of six single fractions (single dose: 0.5 Gy or 1.0 Gy) delivered over three weeks with an interfractional radiation-free interval of at least two days (total dose: 3 Gy or 6 Gy). Patients not showing an improvement of their pain or not being subjectively satisfied six weeks after the end of the first series underwent a consecutive second series 12 weeks following the first. The application of further series occurred only on an individual basis.

#### Mice

Similar to the patient treatment, mice received 0.5 Gy as a locally applied single dose using orthovoltage technique with a X-Strahl orthovolt X-ray machine (180 keV, 10mA, 1mm copper filter) as previously described in (19).

### Measurement of Therapeutic Outcome

The data on patients outcome was collected prospectively exclusively for clinical routine and afterward analyzed retrospectively. The retrospective use of the patient`s data is covered by an approval by the Ethics Committee of the Friedrich-Alexander-Universität Erlangen-Nürnberg (Ref.169_21 Bc, FootRetroRad trial). The study was performed in accordance with the 1964 Declaration of Helsinki and its later amendments. The outcome in the present study was measured by quantifying the subjective pain reduction in terms of percentage of improvement with reference to their initial pain status before LDRT. The interrogation of patients was performed directly after the last radiotherapy session and during the follow-up appointments 8 to 12 weeks later, as well as six months after LDRT. Joints of the foot were discriminated as such: metatarsophalangeal, tarsometatarsal, tarsi-transversa, and the upper and lower ankle joint. The discrimination occurred according to diagnosis of the orthopedic specialist and clinical examination and marking before radiotherapy. Analyses were performed with the subjective rating obtained at the last session of RT, three months post RT and six months post RT, if available.

### K/BxN Mouse Model and Arthritis Scoring

Mice were maintained in a SPF facility under sterile atmosphere at the animal facility of the Universitätsklinikum Erlangen. The animal procedures have been approved by the “Regierung of Unterfranken” and were conducted in accordance with the guidelines of the Federation of European Laboratory Animal Science Associations (FELASA; Approval numbers: 55.2-DMS-2532-2-114 from 13 November 2015 and 10 December 2015; TS-3/14 from 21 February 2014). Pooled serum from transgenic K/BxN mice (29, 38) was kindly provided by F.Nimmerjahn. 200µl serum was injected intraperitoneally into 10 week old female C57Bl/6 mice (Janvier, Le Genest-Saint-Isle, France). Arthritis scoring was carried out in a blinded manner with a score system ranging from 0 (no swelling) to 3 (massive swelling) as described previously (38).

### Flow Cytometry Analysis

For multicolor flow cytometry analysis whole blood and bone marrow (BM) of the mice was collected. Erythrocytes in 100µl whole blood were lysed using a TQ-Prep Workstation (Beckmann Coulter, Brea, CA, USA), for analysis of BM, 1x10^5^ cells/stain were used. Cells (lysed blood cells and BM) were resuspended in 50µl Fc-block solution (eBioscience, San Diego, CA, USA) and incubated for 10min at room temperature. Staining for antibody panels was then carried out at 4°C for 30min as described previously (39). Cells were analyzed using a CytoFLEX S flow cytometer and data was analyzed with the help of the Kaluza analysis software (both: Beckman Coulter, Brea, CA, USA).

### MSD^®^ Multi-Spot Assay System

Multiplex ELISA was carried out with Meso Scale Discovery^®^ system (MSD^®^; Rockville, MD, USA) according to the manufacturer’s recommendation. For this experiment, the Proinflammatory Panel 1 (mouse) Kit V-Plex^®^ Assay (including detection of IFN-γ, IL1-β, IL-2, IL-4, IL-5, IL-6, KC/GRO, IL-10, IL12p70, TNF-α) as well as the TH17 Panel 1 (mouse) Kit (MIP-3α, IL-22, IL-23, IL-17C, IL-31, IL-21, IL-16, IL-17A, IL17E/IL-25) were used. For analysis, serum samples were diluted 1:2 for the Proinflammatory and 1:4 for the TH17 Panel, with the provided buffer respectively, as recommended by the manufacturer. Subsequent analysis was carried out using MSD^®^ Discovery Workbench^®^.

### Statistical Analysis

#### Patient Studies

The Wilcoxon (α = 0.05) test was used to evaluate the difference between groups on the best improvement during therapy using ggplot2 (version 3.3.5) (p<0.05). Univariate and multivariate logistic regression was used to evaluate the association of clinical factors and the best improvement. A correlation analysis of the factors in each group was performed by ggcorrplot (version 0.1.3). Data management was performed using the IBM SPSS software for MS Windows (SPSS Inc Chicago, IL, USA, version 21). All analyses were carried out using R version 3.6.1 (R Foundation for Statistical Computing) and related packages. P-values ≤ 0.05 were considered statistically significant.

#### Pre-Clinical Studies

Data is presented as either Mean ± SEM or Median + IQR. Samples were tested for normal distribution and variance equality and subsequently analyzed using two-tailed Mann-Whitney-U test in comparison to untreated controls. Generation of graphs as well as statistical analysis was done using GraphPad Prism Software (Version 8.3.0; GraphPad software, Inc., San Diego, CA, USA). P-values ≤ 0.05 were considered to be statistically significant.

## Results

### LDRT Improves Pain Levels in Patients

**Figure 1:**
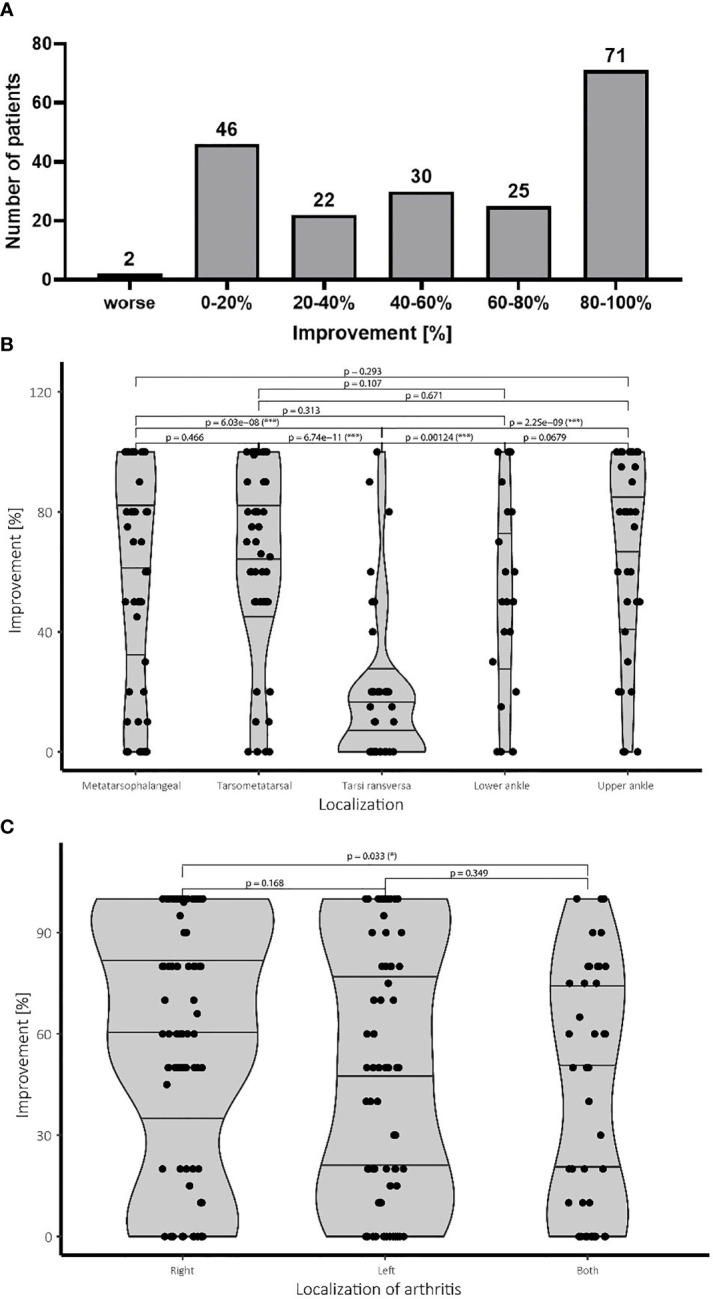
Pain improvement and treatment success following LDRT. **(A)** Patients scored the subjective improvement of their pain level in percentage of improvement in regard of their initial pain level before therapy. The pain levels were determined after the last LDRT session, as well as 12 and 24 weeks after LDRT, if available. The figure depicts the best therapeutic response of the patients independent of the time point and the single and cumulative dose. Patients reporting improvement up to 20% were considered as stable disease. **(B)** Affection of the tarsi transversa region is associated with significantly worse outcome compared to all other localisations. All other treatment groups show a more favorable treatment outcome. **(C)** While treatment response for an affection of the right side is significantly better, affection of the left side does not show worse treatment result than an involvement of both feet. N=196 patients.

The patients scored their subjective pain reduction directly after the last RT session and, if available, after three and six months post treatment. In order to depict the therapeutic success, the best therapeutic response independent of the time point of data collection and the cumulative dose is presented in **Figure 1A**.

The majority of the patients (75%; 148/196 patients) reported a subjective improvement exceeding the clinical benchmark of 20% which was considered as a therapeutic success. An improvement between 20-40% was observed in 11% (22/196) of all patients. Over 15% (30/196) of the patients reported halving of subjective pain reporting improvement of 40-60% whereas 12% (25/196) even reported improvement of around 60-80%. The biggest group of the patient cohort of about 37% (71/196) of the patients even scored near complete and complete response reporting improvement of 80-100%. Only two patients reported a worsening of symptoms.

### LDRT Treatment Success Is Partially Dependent on Localization

Several different confounding factors like age, gender, number of radiation series, location of arthritis, laterality as well as the single dose were examined for their effect on the therapeutic success. For this reason, a univariate and multivariate analysis was carried out. As a cut off, an improvement of 20% was set for the statistical analysis with the best response (independent of the time point of data collection directly after LDRT as well as after 3 month or 6 months post treatment) used for measurement. No correlation between gender and number of series, as well as the applied single dose was observed. A significant impact was found for localization of the OA (see **Figure 1B**) as well as for the age (≥50 years vs. <50 years). Due to these results more in depth investigations were carried out. Results of the univariate and multivariate analysis are depicted in **Table 2**.

**Table 2:** Overview of the results of the univariate and multivariate analysis.

Parameter	Univariate regression			Multivariate regression		
	RR	95% CI	p-value	RR	95% CI	p-value
Gender (male vs. female)	0.619	0.22 – 1.323	0.204	0.893	0.763 – 1.076	0.244
Number of series	0.890	0.386 – 2.010	0.779	1.031	0.851 – 1.249	0.757
Single dose (1.0 vs. 0.5 Gy)	0.668	0.086 – 7.038	0.715	0.962	0.547 – 1.629	0.888
Location (tarsi transversa)	1.087	0.858 – 1.389	0.495	1.020	0.965 – 1.077	0.483
Location (both feet vs. right or left)	0.536	0.251 – 1.179	0.111	0.827	0.670 – 1.014	0.075
Age (≥50 years vs. < 50 years)	5.654	1.541 – 23.052	**0.010**	1.991	1.261 – 3.380	**0.006**

RR, relative risk; CI, confidence interval.

Bold values indicate statistically relevant values.

Patients with affection of the joints of the tarsi transversa show significantly worse treatment response than any other group with the majority of the patients reporting lower levels of improvement. No statistical difference between the other groups concerning treatment success was observed. Despite not being statistically significant the lower ankle seems to show a slight tendency towards a worse treatment result than the upper ankle. Meanwhile an affection of the midfoot at the tarsometatarsal joint shows similar treatment results compared to the forefoot represented by the metatarsophalangeal joints. Summarizing LDRT in the treatment of OA of the tarsi transversa does not seem to be equally effective compared to other localizations of the foot, but should still be considered due to the complete remission achieved in some patients in our cohort as can be seen in **Figure 1B**.

### LDRT Treatment Is Dependent on Laterality

Regarding the side of the affection, significantly worse results were observed for patients suffering from bilateral affection compared to patients suffering only of OA in the right foot. No difference in improvement between the left and right leg as well as the left side and both sides was observed. Therefore, a clearly worse prognosis for affection of both feet cannot be given, although a slightly worse treatment response cannot be ruled out either (**Figure 1C**).

### An Increased Number of Series Does Not Significantly Improve Therapy Outcome

**Figure 2:**
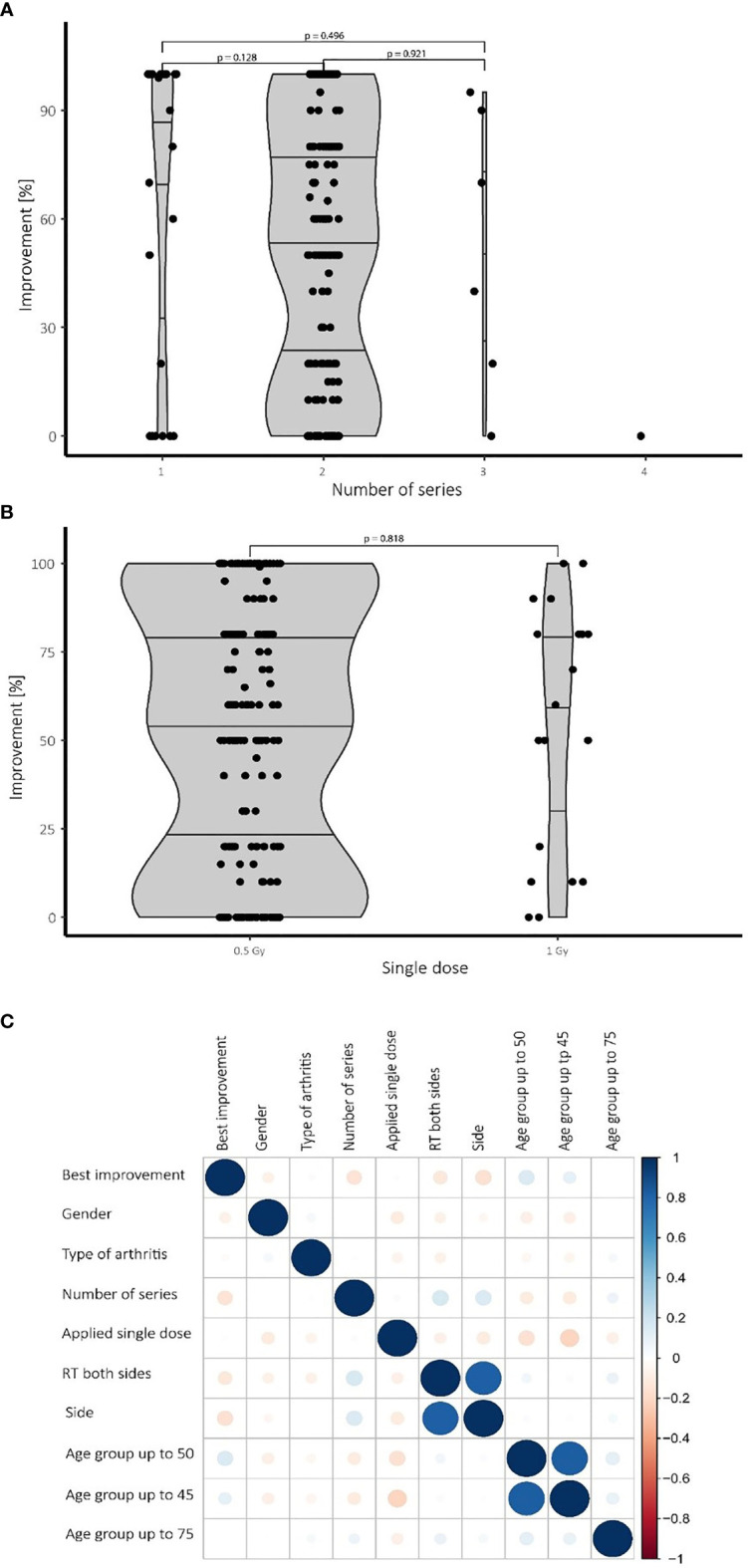
Therapeutic success depending on the number of series and applied single doses also depending on confounding factors. **(A)** While most patients received two series of LDRT, no significant differences in treatment effects have been observed for a higher number of series. **(B)** No significant difference in treatment outcome was observed comparing the 0.5 Gy and the 1.0 Gy group. **(C)** Heat map visualizing various confounding factors: Blue color symbolizes positive correlations while red dots represent negative connections. The size of the dots is proportional to the strength of the correlation. Younger patients (up to 50 years), for example, tend to show a better improvement and need fewer series than older patients. N=196 patients.

While most patients in our cohort received 2 series of LDRT, Some patients demanded further treatment series. Patients receiving a single series of LDRT report a high median of over 60% of improvement compared to pre-therapeutic base line, while also showing a very broad spread between 15% and 100%. Patients undergoing a second or even a third series show a very similar median of improvement compared with patients with two series showing a median improvement of 55% and patients with a third series showing a median improvement of 57% (**Figure 2A**). Despite not being significant, patients undergoing a third series show a small tendency towards a better median and a broader spread towards higher levels of improvement. Only one patient underwent a fourth series.

### A Single Dose of 1.0 Gy Shows No Significant Therapeutic Improvement Over a Single Dose of 0.5 Gy

Neither in the univariate nor in the multivariate analysis nor in the Wilcoxon test significant differences were observed by comparing treatments with a single dose of 1.0 Gy with those of 0.5 Gy. While the 1.0 Gy group shows a slightly higher median, the interquartile distance shows no convincing tendency towards better results of improvement. Despite the lack of a discernible difference, the results (see **Figure 2B**) are still limited by the small number of patients undergoing LDRT with a single dosage of 1.0 Gy.

For a summarizing overview a heat map was created to investigate the impact of confounding factors and the best improvement by logistic regression. Confounding factors include gender, number of radiation series, age, type of arthritis and side of involvement. **Figure 2C** summarizes and visualizes these correlations.

### Locally Applied LDRT With 0.5 Gy Shows Systemic Effects in the Bone Marrow of the K/BxN Serum Transfer Mouse Model

**Figure 3:**
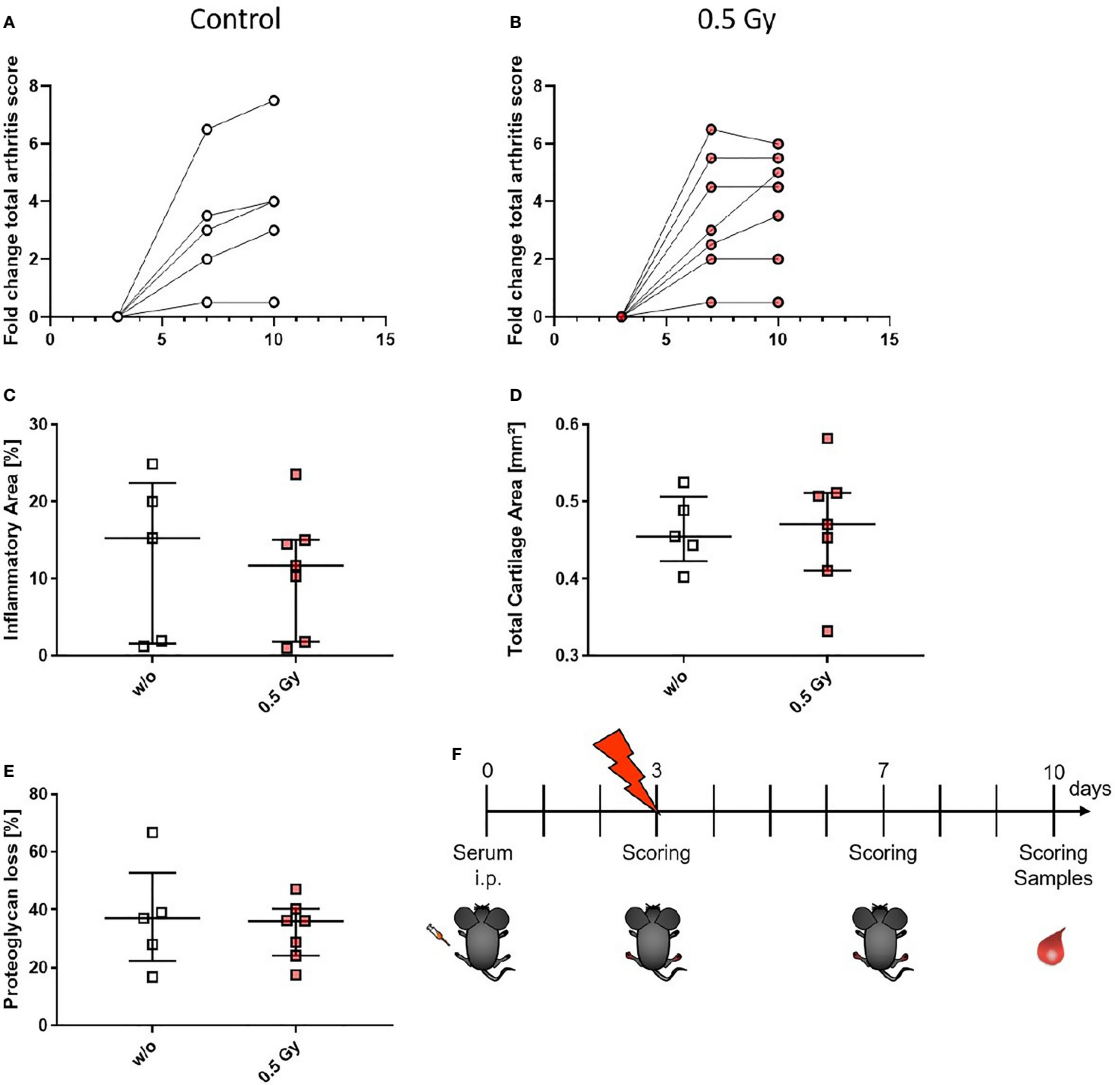
LDRT with 0.5 Gy stabilizes the arthritis score and slightly reduces the inflammatory area in K/BxN serum transfer mice. Age and sex matched mice were injected intraperitoneal (i.p.) with 200µl of K/BxN serum and either locally irradiated with 0.5 Gy X-rays or mock treated on day 3 after the injection (nw/o=5; n0.5Gy=7). Over the entire experimental procedure, arthritis score was assessed **(A, B)**. 7 days after the irradiation hind legs of treated and untreated animals were collected, decalcified, paraffin embedded and cut into 1µm thin slices. Histomorphological evaluation was carried out using the OsteoMeasure™ system (OsteoMetrics, Decatur, GA, USA) after staining with hematoxylin eosin for inflammatory areas **(C)** and toluidinblue for cartilage areas **(D)** and proteoglycan loss **(E)**. **(F)** Shows timeline of pre-clinical experiments: Female C57Bl/6 mice were injected i.p. on d0. On d3 after the injection, mice were scored and randomly distributed into two groups. While one group received mock treatment, the other received 0.5 Gy of locally applied X-rays. Both groups were scored again on d7 and d10. On d10 mice were sacrificed and samples (whole blood, serum, bone marrow and hind feet) were collected. Data is presented as Median+IQR.

**Figure 4:**
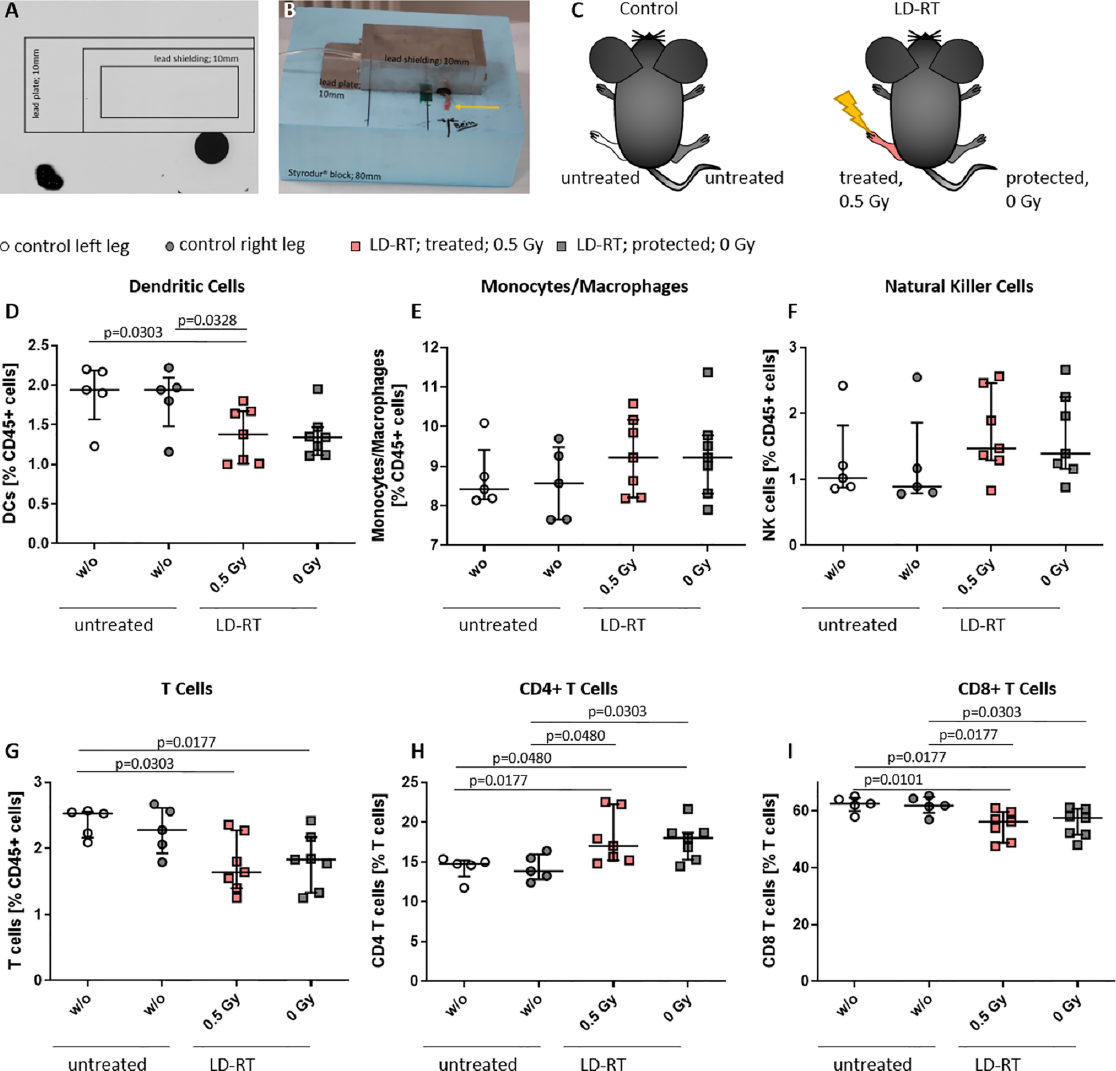
Locally applied LDRT with 0.5 Gy systemically alters distinct immune cell subsets in the bone marrow of treated and protected legs of K/BxN serum transfer mice. Age- and sex-matched mice were injected i.p. with 200µl of K/BxN serum and either locally irradiated with 0.5 Gy X-rays or mock treated on day 3 after the injection (nw/o=5; n0.5Gy=7) **(A–C)**. 7 days after the irradiation, bone marrow was collected and flow cytometry analysis was carried out for the indicated immune cell subtypes **(D–I)**. The figure shows different immune cell subsets as found in the left or right hind leg of mock treated (control left/right leg) and irradiated animals (treated 0.5 Gy or protected 0 Gy) **(C)**, respectively. Data is presented as Median + IQR, statistical analysis was carried out using Mann-Whitney U test, statistical significances are indicated above.

As these studies are performed retrospectively and researchers are often confronted with the worries of these effects being a placebo effect, we aimed to look into the molecular effects behind the analgesic properties of LDRT by using a pre-clinical model system. K/BxN serum transfer mice were locally irradiated with a local single dose of 0.5 Gy [**Figures 3**, **4**, and as previously described in (19)]. While assessment of clinical score in the mice pointed towards a similar response as in patients (e.g. incline in disease progression in untreated and stable or declining disease in 5/7 of the treated animals 1 week after LDRT; **Figures 3A, B**), histomorphological effects of the LDRT treatment displayed a slight reduction in the inflammatory area alongside a slight tendency of increased cartilage area (**Figures 3C, D**). When analyzing bone marrow cells, derived from the long bones of the hindfeet of mock and LDRT treated animals, we found that distinct immune cells were significantly systemically altered in LDRT-treated mice (**Figures 4D–I**).

Among the most affected cell types were dendritic cells (DCs) that were found to be significantly reduced in the irradiated leg of treated animals (**Figure 4D**). In the case of T cells, a general significant reduction of total T cell numbers in both legs (treated and protected) of the irradiated animals (**Figure 4G**) was detected. Notably, T cell subsets shifted from the rather inflammatory CD8+ T cells, that were found to be significantly reduced in both legs of the LDRT-treated animals (**Figure 4I**) to CD4+ T cells that, depending on the subtype, can have rather anti-inflammatory effects, and that were found to be significantly increased in both legs, respectively (**Figure 4H**).

### LDRT With 0.5 Gy Only Slightly Modulates Immune Cell Subsets and Their Activation Status in the Peripheral Blood of K/BxN Serum Transfer Mice

**Figure 5:**
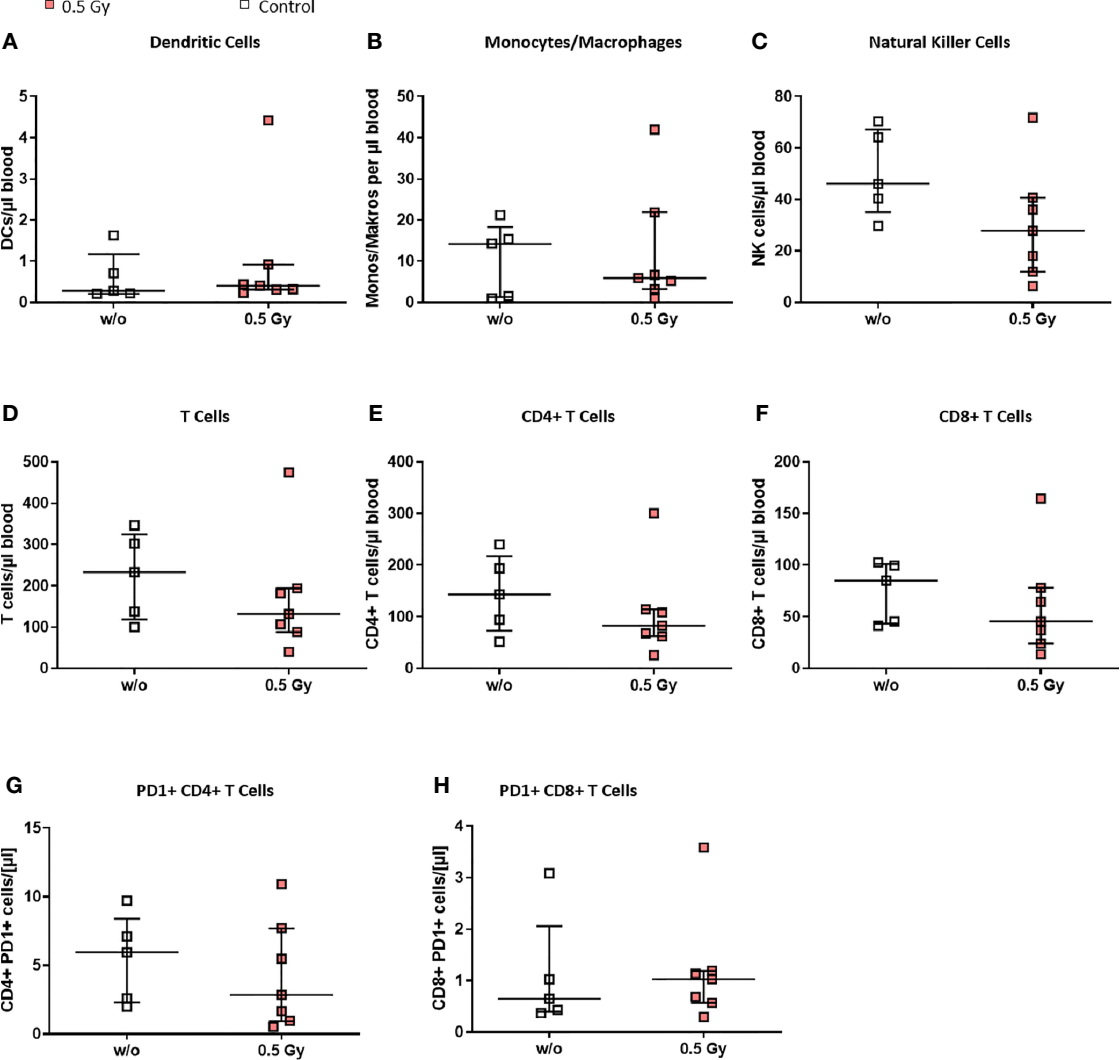
LDRT with 0.5 Gy induces slight changes of immune cells in the peripheral blood of K/BxN serum transfer mice. Age and sex matched mice were injected i.p. with 200µl of K/BxN serum and either locally irradiated with 0.5 Gy X-rays or mock treated on day 3 after the injection (nw/o=5; n0.5Gy=7). 7 days after, the whole blood of all animals was collected, erythrocytes were lysed and flow cytometry analysis for immune cell subtypes **(A–H)** was carried out. Depicted are different immune cell subsets as found in the whole blood of mock treated (w/o) and irradiated animals (0.5 Gy), respectively. Data is presented as Median + IQR, statistical analysis was carried out using Mann-Whitney U test.

We then wanted to elucidate whether the effects observed in both legs of the LDRT treated mice could be explained through a generally systemic response, and therefore examined the peripheral blood of the animals *via* flow cytometry (see **Figure 5**). Monocytes/macrophages (**Figure 5B**) and NK cells (**Figure 5C**) tended to be lower in LDRT treated animals in comparison to mock treated ones. In addition, similar to the findings in the BM (**Figure 4**), T cell numbers in LDRT treated animals were reduced (**Figure 5D**), while no shift from CD8+ (**Figure 5F**) to CD4+ T cells (**Figure 5E**) could be observed in the peripheral blood, as both T cell subsets were found to be generally decreased after LDRT.

Notably, we found slightly increased numbers of exhausted CD8+ PD-1+ T cells in LDRT treated animals (**Figure 5H**), while the numbers of exhausted CD4+ PD-1+ T cells were found to slightly decrease after LDRT (**Figure 5G**).

### Locally Applied LDRT Results in an Anti-Inflammatory Cytokine Milieu in K/BxN Serum Transfer Mice

**Figure 6:**
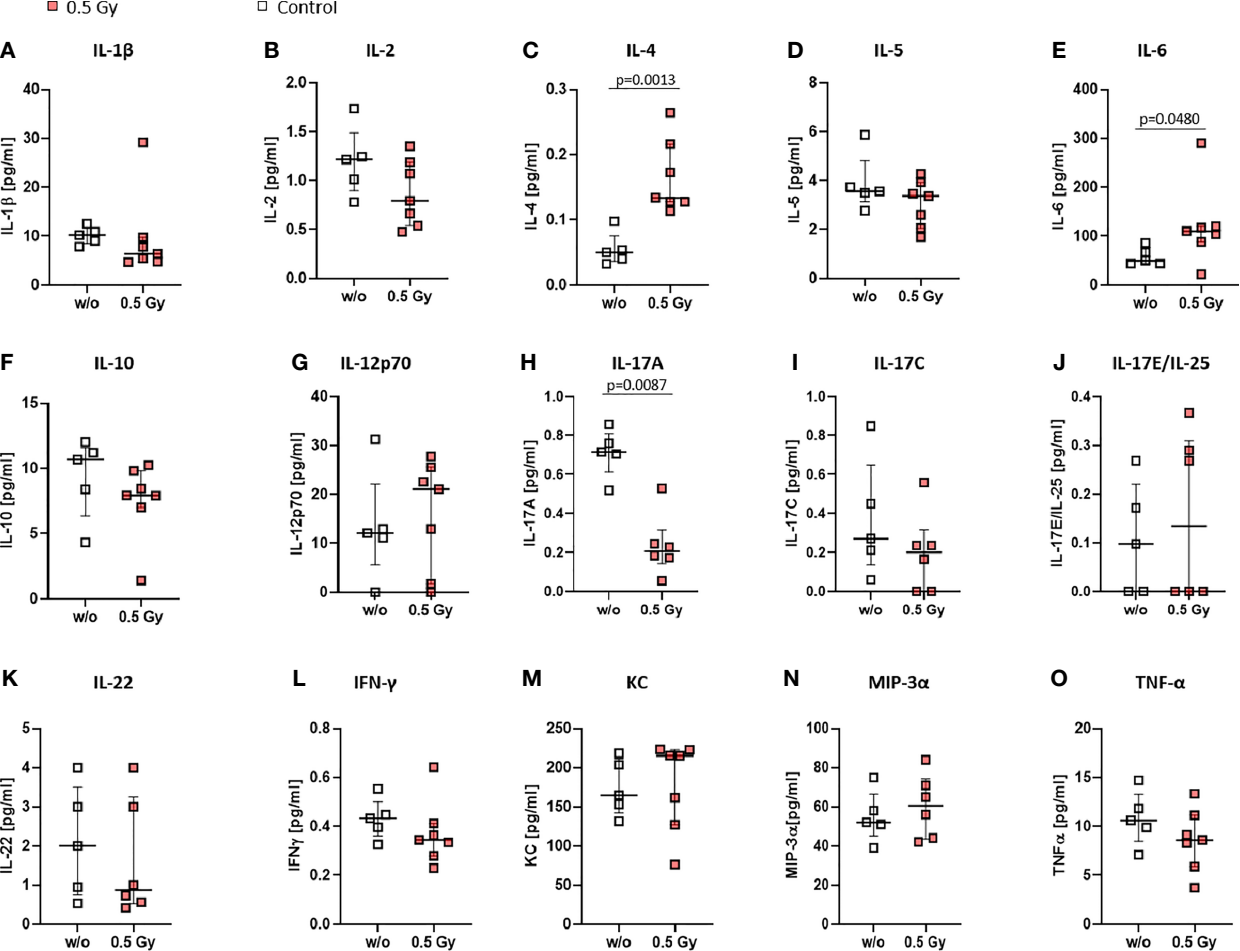
Locally applied LDRT with 0.5 Gy affects pro-inflammatory cytokines in K/BxN serum transfer mice. Age and sex matched mice were injected i.p. with 200µl of K/BxN serum and either locally irradiated with 0.5 Gy X-rays or mock treated on day 3 after the injection (nw/o=5; n0.5Gy=7). On day 7 after the irradiation, serum was collected and analyzed via MSD Multiplex Assay **(A-–O)**. Depicted are serum levels of various cytokines playing a role in inflammatory processes **(A–O)**. Data is presented as Median + IQR, statistical analysis was carried out using Mann-Whitney U test, statistical significances are indicated above.

As alterations in immune cell subsets in both, bone marrow and peripheral blood, were observed following LDRT, we then looked into serum levels of various cytokines to examine whether altered immune cell subsets could be due to altered cytokine milieu (**Figure 6**). Most of the examined cytokines were generally found to be slightly altered after LDRT (decrease: Interleukin (IL)-1β, IL-2, IL-10, IL-17C, IL-22, Interferon (IFN)-γ, Tumor necrosis factor (TNF)-α; increase: IL-4, IL-6, IL12p70, IL-17E/IL-25 KC, MIP-3α; no alteration: IL-5) when being compared to serum levels of mock treated control animals. However, significantly increased serum levels were found for IL-4 (**Figure 6C**) and IL-6 (**Figure 6E**), while IL-17A levels were found to be significantly decreased (**Figure 6H**) after LDRT.

## Discussion

The use of LDRT has often been offered as an alternative treatment option in the treatment of OA and other degenerative joint diseases if first line treatments have failed. This is due to the positive results achieved and the vast body of data attesting to its efficacy (40–43). In the treatment application orthovoltage as well as megavoltage radiotherapy techniques have been used successfully. Despite recent prospective data showing disappointing results, several limitations of these studies like the short-term follow-up of only 3 months, low patient numbers, poor patient selection, reduced treatment volume and too optimistic prognosis assessment for the evaluation of treatment success have to be taken into account (44–47). Data investigating the effect of LDRT on osteoarthritis of the foot have been published before (37, 40). The presented data of our analyses investigates a larger number of patients, and performs detailed correlation of localization and treatment success. As a further difference to previous studies, the present cohort of patients has homogenously undergone orthovoltage therapy. The analysis of 196 patients confirms the convincing data of LDRT in the treatment of osteoarthritis of the ankle and foot with a majority of patients reporting improvement right after radiation. With only 1% (2/196) of the patients reporting a worsening of symptom burden following radiation and only 25% (48/196) patients reporting improvement <20%, the rate of non-responders has to be regarded as low. Regarding the fact that LDRT is commonly applied for patients that are refractory to classical treatment strategies, this finding is even more promising.

Investigating the success of treatment depending on localization, clearly worse treatment results can be expected for patients suffering from OA of the tarsi transversa. Despite this exact localization not being overrepresented in our cohort and in general thought to be rather uncommon, the involvement seems to be associated with a worse prognosis. While the genuine reasons remain unknown due to the retrospective approach of the present study one possible explanation could be related to previously experienced injuries. While the joint is not only of significant importance during the stance phase but also of utmost importance for pivoting movements, injuries can involve high as well as low energy forces. Mechanism of injury usually involves twisting force to the plantar flexed foot requiring carful diagnosis and high-quality radiographs (48). The high rates of up to 40% of misdiagnosis could therefore lead to long term chronic impairment (48). Another explanation could be due to the joint of the tarsi transversa showing the highest maximum joint reaction forces among all joints of the foot, potentially hindering recovery (49).

While a large number of investigations using classifications of forefoot, midfoot and hindfoot have been carried out, very few data is published on the affection of the side of the feet. Our results show no difference in treatment success between the right and the left foot, while patients with symptoms of the right foot seem to respond better to treatment compared to patients suffering symptoms of both feet. One possible explanation could be seen in the fact that in most patients the right foot is the dominant foot and thus more prone to acute inflammatory conditions that are more addressable by LDRT, while affection of both feet could possibly occur due to more degenerative processes. The lack of significance for the left foot could possibly be due to a combination of inflammatory and degenerative processes.

Regarding the number of applied series, no tendency towards improved results for a higher number of series can be detected in our cohort. While the application of two series is most common, the request for further series, which are applied only on an individual basis and on personal request, implies that patients may experience worsening of symptoms following a good initial response and strive towards recovery of their initial treatment success. Although the application of a third series does not significantly improve treatment outcome the results at least suggest that a recovery after initial worsening seems to be possible. Apart from the very small number of patients in this cohort, our data does not support the application of a fourth series. Despite no side effects being reported in our cohort the benefit of additional LDRT series should be discussed with the patient and balanced against the potential radiation risk (50). While there is a vast body of data showing that a single dose of 0.5 Gy is superior to 1.0 Gy regarding anti-inflammatory effects, our data also does not support the application of a single dose per fraction of 1.0 Gy, despite being often utilized and still embedded in guidelines (19, 51).

While OA is known to be a disease of more advanced age, it is even more impressive and a novel result to see that younger patients show better improvement (**Table 2**). Younger patients might suffer more from inflammatory conditions rather than advanced degenerative conditions which are more addressable by LDRT.

As radiobiological data recommends, we used a single dose of 0.5 Gy for our animal experiments. While in our patient study neither dosage showed superiority over the other, in general, a reduced dosage is always advisable, especially in foresight of potential additional LDRT series, as mentioned above. In previous studies (19) we already showed that in a human TNF-α transgenic mouse model, local irradiation with 0.5 Gy leads to an improvement of inflammatory areas not only in the irradiated but also in distant extremities, while bone erosions were only reduced locally at the site of irradiation. We therefore hypothesize, that there are several mechanisms involving inflammation and bone repair that are responsible for LDRT mediated beneficial effects. This is also visible in the examined patient cohort, as most patients report a reduction in pain levels, while data does not show improvement in all of the joints. We therefore aimed to look into immunological modifications after LDRT in a K/BxN serum transfer mouse model, in order to better understand modulation of inflammation after LDRT which could be related to improved pain perception in patients.

While OA is generally looked at as a non-inflammatory disease, increasing evidence points towards inflammatory processes at the site of bone erosions as well. In that matter, OA patients often show infiltration of the synovial membrane by immune cells such as e.g. macrophages, T cells, B cells natural killer cells (NKs), and DCs, all of these immune cells can also be found in the mouse model (29, 52).

When looking into the BM, we found a significant reduction of DCs in the irradiated legs of locally irradiated mice (see **Figure 4**). While the role of DCs in OA is not yet fully understood, inflammatory DCs are involved in OA inflammation and inhibition of chondrogenesis (53). While significant alterations were found in the BM, no significant differences between LDRT and mock treated animals was found in the blood (**Figure 5**). However, DCs are able to pass on either stimulatory or anti-inflammatory signals to T cells and B cells (54), in the form of cytokines. In the case of T cells, IL-12p70 for the induction of T_H_1, IL-4 for T_H_2, and IL-17 for T_H_17 cells (55), are of special interest. As can be seen in the cytokine profile in the serum of LDRT treated mice, IL-12p70 was found to be slightly, and IL-4 to be significantly increased after LDRT in the serum of the mice (**Figures 6C, G**). This potentially indicates an increased T_H_1 and T_H_2 response, while IL-17A levels were significantly reduced (**Figure 6H**), suggesting a reduced number or activity of T_H_17 cells. While T_H_17 cells are predominantly looked at as target cells in RA, there are also indications of T_H_17 cells being elevated in OA lesions as well (52). Reduced amounts of IL-17A might thus contribute to an overall anti-inflammatory response in LDRT treated animals.

While total T cell numbers were reduced in the BM (**Figure 4**) and blood (**Figure 5**), a shift from CD8+ in favor of CD4+ T cells was only visible in the BM. While CD8+ T cells have not been found to be key players in OA, they have been found to be able to significantly shape OA pathogenesis (52). A reduction in CD8+ and increase of CD4+ T cells might therefore add to the overall beneficial anti-inflammatory and analgesic effects of LDRT in OA. While these differences were not found in the peripheral blood of treated animals, we did find a slight increase in CD8+ PD-1+ T cells, while CD4+ PD-1+ T cells were decreased (**Figure 5**). T cell exhaustion is characterized by increased amounts of inhibitory surface receptors, one of them being PD-1 (56). This finding thus points towards a potential inhibition of cytotoxic T cells, while the activity of CD4+ T cells is not compromised by LDRT.

In general, differences in immune cell subsets in the peripheral blood were lower than those in the BM. A potential explanation for alterations of immune cell subsets in the periphery is the influence of an altered cytokine milieu. Indeed, we also found alterations in the cytokine milieu in the serum of treated animals (see **Figure 6**). While, in general, pro-inflammatory cytokines were found to be slightly reduced after LDRT with 1x 0.5 Gy, IL-6 (**Figure 6**) on the other hand was found to be significantly increased. IL-6 is generally looked at as a pro-inflammatory cytokine that is involved in a plethora of pathways, one of them being bone metabolism. While it mainly has pro-resorptive and -inflammatory properties, under certain conditions it can also show anti-resorptive effects on bone through e.g. an increase in osteprotegerin, a potent inhibitor of osteoclastogenesis (57). However, further research is needed in elucidating the effects of IL-6 in this specific setting.

Among the most prominent alterations is IL-4 that was found to be significantly upregulated after LDRT (**Figure 6**). IL-4 is involved in the induction of T_H_2 cells, enhances IL-10 production in T_H_1 cells, as well as polarizes macrophages towards a more anti-inflammatory phenotype. Additionally, it has been found to have antagonistic effects in OA with a special emphasis on chondroprotective effects, which, however, where not visible in the short observation period following LDRT (**Figures 3D, E**). In that matter Wojdasiewicz et al. summarized that it was found to inhibit degradation of proteoglycans through an inhibition of matrixmetalloproteinases (58). Our findings thus suggest a potential beneficial effect of LDRT on cartilage degradation in OA at later timepoints.

Summarizing our study, some limitations have to be addressed: While selecting radiological findings or questionnaires as endpoint seems intuitive for the measurement of the clinical effect, these exact scales haven not proven valid and sensitive for the recording of the efficacy of LDRT in previous studies (44, 45). Nevertheless one point that needs to be addressed in future studies is the severity of OA. Different grades of OA should be correlated to treatment results to increase informative value. After all we are convinced that biomarkers can be a lot more sensitive. While in the present study patients were referred for LDRT mostly by their orthopedic specialist after failing several preceding treatment approaches, the time interval between initial imaging and actual treatment could have varied a lot between patients which is why we chose not to evaluate initial imaging. For future prospective studies if imaging is intended we would recommend imaging right before treatment especially using MRI as this is the best modality to capture anti-inflammatory changes. Despite functionality tests being outside the scope of the present study they could underline the clinical significance to this treatment method in a prospective setting. Including patient logbooks reporting on medication intake and pain perception could further help to distinguish LDRT-mediated effects from medication related improvement in order to reduce bias in data interpretation and need to be subject in prospective trials. Regarding our pre-clinical data, we have to stress, that neither this, nor an alternative available OA model, is able to completely resemble the situation in our patient cohort. Especially, since localization and severity of OA can significantly vary between patients. However, as patient material usually is scarce, the present pre-clinical insights could possibly improve future study designs by indicating potential key factors of the involved biological mechanisms. This enables an optimized usage of patient material in future prospective studies.

In conclusion, LDRT is highly effective in the majority of patients undergoing treatment for OA of the foot and ankle. While clinically acquired data are justifiably predominantly utilizing clinical questionnaires and radiographical signs, the underlying molecular mechanisms have not been investigated sufficiently. Our results clearly show local as well as systemic effects in a mouse model, which could be responsible for the beneficial clinical results achieved by LDRT. One can further conclude, that the observed anti-inflammatory and analgesic effects of LDRT are not mediated by a single pathway, but rather by the interplay of many individual factors. Nevertheless, further pre-clinical research as well as placebo-controlled patient studies are necessary in order to fully understand the underlying mechanisms Our results should serve as a basis for well-reasoned and carefully planned placebo-controlled patient studies in the future. By improving the understanding of the underlying molecular processes and presenting our convincing clinical results, we hope to encourage more clinicians to rely more on this low risk and cost effective treatment option.

## Data Availability Statement

The data presented in this study are available from the corresponding author on reasonable request.

## Ethics Statement

The studies involving human participants were reviewed and approved by Ethics Committee of the Friedrich-Alexander-Universität Erlangen-Nürnberg. Written informed consent was obtained from all patients before LDRT. In addition, patients provided written informed consent for the retrospective analysis of their data, as well as the publication of the data in a pseudonymized manner (ethical approval Ref.169_21 Bc, FootRetroRad trial). The animal procedures have been reviewed and approved by the “Regierung of Unterfranken” and were conducted in accordance with the guidelines of the Federation of European Laboratory Animal Science Associations (FELASA) Approval numbers: 55.2-DMS-2532-2-114 from 13 November 2015 and 10 December 2015; TS-3/14 from 21 February 2014).

## Author Contributions

TW, LD, and UG designed the study and wrote the manuscript. TW analyzed patient data, LD performed and analyzed pre-clinical data, MR, A-JD, and MS helped performing the experiments. J-GZ performed the statistical analysis of patient data. FN helped with the design of K/BxN model experiments and contributed to writing the manuscript. BF designed the *in vivo* irradiation chamber and performed irradiation of mice. RF contributed to the experimental design and writing of the manuscript. SL, OO, FP, and MH critically revised the manuscript and contributed to writing the article. All authors contributed to the article and approved the submitted version.

## Funding

This work was funded by the *Bundesministerium für Bildung und Forschung* (BMBF; GREWIS, 02NUK017G, GREWIS-alpha, 02NUK050E; as well as FOR 2886) as well as the German Research Foundation (DFG; Research Training Group GK16660 as well as CRC1181-A07).

## Conflict of Interest

MH conflict of interest with Merck Serono (advisory role, speakers’ bureau, honoraria, travel expenses, research funding); MSD (advisory role, speakers’ bureau, honoraria, travel expenses, research funding); AstraZeneca (research funding); Novartis (research funding); BMS (advisory role, honoraria, speakers’ bureau); Teva (travel expenses). RF conflict of interest Astra-Zeneca and MSD as well as honoraria from Merck Serono, Astra-Zeneca, MSD, Novocure and research funding from Fresenius Nutrition, Merck Oncology, MSD, Astra-Zeneca, Novocure and Siemens AG. FP reports grants and speaker fees from Siemens Healthcare GmbH not related to the present work.

The remaining authors declare that the research was conducted in the absence of any commercial or financial relationships that could be construed as a potential conflict of interest.

## Publisher’s Note

All claims expressed in this article are solely those of the authors and do not necessarily represent those of their affiliated organizations, or those of the publisher, the editors and the reviewers. Any product that may be evaluated in this article, or claim that may be made by its manufacturer, is not guaranteed or endorsed by the publisher.
